# Evaluating the impact of interactive video-based case-based learning in clinical medical education: a randomized controlled trial

**DOI:** 10.3389/fmed.2025.1556018

**Published:** 2025-05-16

**Authors:** Jing Wang, Yang Jiang, Xinghua Fu, Ruiqiang Gou, Zhijing Sun, Ge Li, Wei Zhang, Jin Nie, Wenling Wang, Kun Zhao, Li Wang, Ruihong Zhang

**Affiliations:** ^1^School of Public Health, Peking University, Beijing, China; ^2^Jitang College, North China University of Science and Technology, Tangshan, Hebei, China; ^3^The Fourth Affiliated Hospital of Harbin Medical University, Harbin, Heilongjiang, China; ^4^The First College of Clinic Medicine, Lanzhou University, Lanzhou, Gansu, China; ^5^School of Public Health, Southern Medical University, Guangzhou, Guangdong, China; ^6^Shandong University of Traditional Chinese Medicine, Jinan, Shandong, China; ^7^General Surgery Department of Baoding Fourth Central Hospital, Hebei, China; ^8^Obstetrical Department of Baoding Fourth Central Hospital, Hebei, China; ^9^Infection Management Department of Baoding Fourth Central Hospital, Hebei, China; ^10^The First People’s Hospital of Jining, Jining, Shandong, China; ^11^Orthopedics Department of Baoding Fourth Central Hospital, Hebei, China; ^12^The Fourth Central Hospital of Baoding, Hebei, China

**Keywords:** interactive video, case-based learning, clinical medical education, clinical thinking, teaching innovation, randomized controlled trial

## Abstract

**Background:**

Traditional Case-Based Learning (CBL) methods in clinical medical education are often hindered by limitations in scalability and student engagement. In response, interactive video-based CBL integrates decision tree scenarios with interactive technology, offering a novel approach to enhance students’ clinical reasoning and learning outcomes.

**Objective:**

This study aims to evaluate the effectiveness of an interactive video-based CBL teaching method in improving clinical knowledge, thinking ability, course experience and satisfaction among undergraduate medical students.

**Methods:**

A single-center, single-blind, randomized controlled trial was conducted with 64 fourth-year clinical medicine undergraduates, who were randomly assigned to either the intervention group (interactive video-based CBL, *n* = 32) or the control group (traditional CBL, *n* = 32). The primary outcomes included basic knowledge test scores, which were assessed both before and after intervention. Secondary outcomes encompassed clinical thinking abilities (critical thinking, systematic thinking, evidence-based thinking) and course experience, measured using validated scales. Data were analyzed using paired and independent tests.

**Results:**

Sixty-two students completed the study. The intervention group showed significant improvement in post-intervention basic knowledge test scores compared to both their baseline (*P* < 0.001) and the control group (*P* < 0.001). Conversely, the control group showed a significant decline in post-intervention scores (*P* < 0.001). Critical and systematic thinking abilities in the intervention group significantly improved after the intervention (*P* = 0.045 and *P* = 0.048), while no significant changes were observed in the control group. No significant changes were observed in evidence-based thinking. Course experience scores were significantly higher in the intervention group across dimensions including good teaching (*P* = 0.041), classroom quality (*P* = 0.033) and classroom gains (*P* = 0.032). The intervention group was significantly more satisfied than the control group overall (*P* = 0.011).

**Conclusion:**

Interactive video-based CBL significantly enhances basic knowledge, critical thinking, and students’ course experience and satisfaction compared to traditional CBL, highlighting its potential as an innovative teaching method in clinical medical education. Further research is needed to explore its long-term impacts and optimize its application for fostering evidence-based thinking.

**Clinical trial registration:**

https://clinicaltrials.gov/, identifier ChiCTR2300073773.

## 1 Background

With the continuous evolution of global educational models, enhancing the quality and efficiency of medical education has become a central focus of teaching reforms worldwide. Clinical medical education, in particular, emphasizes the development of students’ practical skills and clinical thinking abilities. However, traditional teaching methods, which primarily rely on lecture-based learning, often fail to fully engage students and foster their independent learning and practical application skills (1). In recent years, Case-Based Learning (CBL) has emerged as a prominent teaching method in clinical medicine (2). This approach effectively simulates real medical scenarios, encouraging students to apply their theoretical knowledge to solve practical problems (3–5). It has gained significant popularity in both academic and clinical education circles. The CBL model promotes critical thinking and systematic problem-solving by guiding students through the study of typical cases, thereby enhancing their analytical capabilities (6, 7). A meta-analysis by Cen et al. (8) demonstrated that CBL significantly improves medical students’ academic performance compared to other teaching methods (9). As a result, CBL has become an integral component of modern medical education.

Despite its success in improving clinical thinking skills, the traditional CBL model faces several challenges. The lack of flexibility and accessibility in conventional CBL teaching often limits students’ full participation, which negatively impacts learning outcomes (10). Furthermore, traditional offline teaching methods are insufficient to meet the demands of large-scale education, particularly during emergencies such as pandemics (11), where the limitations of face-to-face teaching become even more apparent. The introduction of the online CBL teaching model addresses these issues. Online CBL utilizes digital platforms and virtual environments to facilitate clinical case discussions with undergraduate students (12). Through this method, students apply theoretical knowledge to clinical practice and engage in in-depth discussions and analysis of real-world cases (13). By leveraging the advantages of online platforms, the online CBL model fosters active learning, which plays a crucial role in developing critical thinking skills (14). Shrivastava et al. (15) highlighted that online CBL was an effective teaching method that enhanced student engagement and supported the application of theoretical knowledge in clinical practice. Similarly, Liu et al. (16) found that online CBL was positively evaluated by both students and facilitators, noting improvements in accessibility and flexibility. The online CBL model provides a platform for continuous interaction and participation, reducing the need for synchronous face-to-face interaction, which in turn improves work and learning efficiency (17, 18).

As online teaching models continue to evolve, interactive video-based teaching has garnered significant attention due to its immersive and engaging learning experiences. Interactive video teaching integrates multimedia resources—such as video, audio, and animation—with interactive features like real-time Q&A, quizzes, and scenario simulations (19). This approach aims to enhance student engagement and learning by actively involving students in the educational process (20). Seckman (21) found that interactive video communication was more effective than text-based feedback in promoting teaching presence, social presence, and cognitive presence, highlighting its potential to improve student engagement in online education. Interactive video teaching provides a multisensory learning experience, which not only improves attention and comprehension but also enables students to better understand and apply the knowledge through activities such as Q&A sessions and scenario simulations (22, 23). This model offers a personalized learning path, accommodating the diverse needs of students (24), and is particularly suited for disciplines such as clinical medicine that require high levels of practicality and application.

Despite its broad potential, research on the integration of interactive video-based teaching with the CBL model remains in its early stages, especially in the field of clinical medicine (25, 26). The combination of interactive videos with CBL not only preserves the inherent interactivity and practicality of the CBL model but also addresses the limitations of traditional teaching methods in large-scale and online education settings. This approach has the potential to become a new model for enhancing the effectiveness of clinical medical teaching. Our study aims to develop and evaluate a CBL teaching model that incorporates interactive videos, using a peptic ulcer course for undergraduate clinical medicine students as a case study. It will assess the impact of this model on students’ clinical knowledge, skills development, clinical thinking abilities, course experience, and overall teaching satisfaction. A randomized controlled trial will compare this new model with the traditional CBL model. Our study seeks to explore the effectiveness of interactive videos in medical education and provide innovative insights and a practical foundation for future medical teaching reforms. This approach not only introduces a novel teaching tool but also establishes the groundwork for the widespread adoption and development of interactive videos in the medical field.

## 2 Materials and Methods

### 2.1 Study design

This study was a single-blind, single-center prospective randomized controlled trial (trial registration number: ChiCTR2300073773), which was approved by the Ethics Committee of the Baoding No. 4 Central Hospital (Approval No. 2023031) prior to the start of the trial. The trial report met the CONSORT reporting criteria, and the inclusion and exclusion criteria were as follows.

The inclusion criteria were:

(1) Aged ≥ 18 years;
(2) Proficient in the use of smartphones.

Exclusion criteria were:

(1) Participated in a trial related to the teaching of CBL through interactive videos of decision tree clinics.
(2) Suffering from serious physical or mental illness.
(3) Not signing the informed consent form.

### 2.2 Sample size

A randomized controlled trial design was used in this study. The intervention group was taught CBL based on interactive video and the control group was taught traditional CBL. The theoretical examination scores of the study participants were the main outcome indicators of the observation. According to the relevant literature (27), the difference value between the experimental group and the control group is 5, and the standard deviation is 4, assuming that the two-sided α = 0.05, the power of 1-β is 0.9, and the sample size ratio between the intervention group and the control group is 1:1; referring to the method of Chow et al. (28), the sample size of the intervention group is calculated by R language. The sample size of the intervention and control group were 14 cases each. Considering that 20% of the patients were lost or refused to follow up, the intervention group and the control group needed at least 18 cases each, and the total sample size was at least 36 cases.

### 2.3 Randomized

**FIGURE 1:**
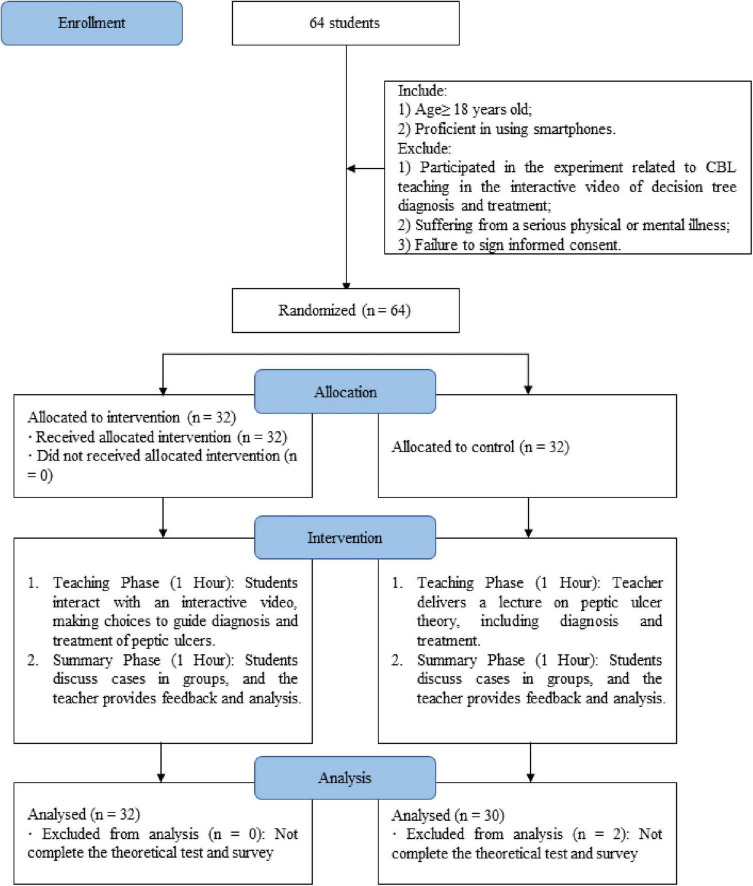
Flowchart of the study.

After all participants voluntarily signed an informed consent form, a random number table method was used to match each participant with an independent random integer, and participants were grouped according to their final digits. Participants with odd end digits were assigned to the interactive video group, while participants with even end digits were assigned to the traditional instruction group. The flowchart of the study is shown below (Figure 1).

### 2.4 Development of interactive video

Based on the training objectives of clinical medicine, the curriculum development team (speciality covering clinical medicine and basic medicine) and the deputy chief physicians (2) selected peptic ulcer disease, and screened cases with typical clinical characteristics from disease risk factors, symptoms, signs, complications, and auxiliary examinations (laboratory tests and imaging tests) to ensure the effectiveness of teaching and training.

The course development team designs the disease development pathway based on the evolution of the clinical manifestations and treatment of the case, anticipates the possible development and evolution of the case under different therapeutic decision-making conditions, and develops a decision tree node, where each node of the decision tree represents a clinical decision point:

(1) The student guesses the possible diseases the patient may have based on his clinical presentation and history and makes decision to prescribe laboratory tests and imaging tests;
(2) The decision to make a diagnosis and consider complications based on the patient’s findings in relation to the etiology of the disease;
(3) Making decisions about treatments based on the patient’s physical condition and illness.

If a student answers a question incorrectly, the decision tree needs to inform the student of the possible consequences and guide the student to redecide until he/she returns to the correct path. Applying decision tree learning helps learners to understand the complete path from conditional judgment to final decision and enhances the knowledge related to decision points.

On the interactive video website.^1^ The designed decision paths and nodes are entered into the website by the course development team, and the website automatically generates interactive videos and links.

At the end of the development phase, five clinical medical students who were not study participants were invited to pre-test and collect relevant comments after which they looked for any deficiencies or problems in the developed interactive video, especially whether there were problems in the order and logic of the presentation of the knowledge points.

### 2.5 Research processes

#### 2.5.1 Unified pre-course teaching program

Before the lesson, the teacher collects disease data and information about peptic ulcer patients according to the syllabus and develops a teaching case for Liu. The case is as follows: Liu, male, 38 years old, employee. The patient developed burning pain in the upper abdomen with nausea and hot air due to improper diet 4 years ago, but no vomiting. The symptoms were relieved after taking gastric medication on his own. Since then, the above symptoms often appeared in the autumn and winter, winter and spring seasons or when he was busy at work. The pain worsens on an empty stomach and subsides after eating. During the attack, there was a slight weight loss, no acid reflux, and no fever. One week ago, due to stress and overwork at work, the above symptoms reappeared and worsened from before, so he came to our hospital for further consultation. He was in good health and denied having traveled to an infected area or having been exposed to infectious diseases. The patient had a 10-year history of smoking.

Several exercises related to peptic ulcer were set up for students to discuss based on the case, including; what are the current diagnostic considerations for this patient? What laboratory and other tests are needed to confirm the diagnosis? The urease test was positive for Hp; what is the preferred treatment medication? How many days of treatment? What is the patient’s current primary diagnosis/problem and basis for it? What is the appropriate treatment? etc.

The instructor distributes the cases and questions to the interactive video group and the traditional teaching group 1 week in advance. Students in each group independently reviewed textbooks, literature, and websites to determine the answers to the questions and develop their own ideas for diagnosis and treatment in preparation for class discussion.

#### 2.5.2 Intervention group

(1) Instructional phase 1 h: students in the interactive video group log on to https://www.bilibili.com/ and open the interactive video on peptic ulcer diagnosis and treatment based on decision trees and scenarios. While watching the video, students interacted with questions that popped up in the video and chose answers to different questions that led to different teaching scenarios. For example, if an initial diagnosis of gastric ulcer is made, the interactive video pops up the causes of gastric ulcer and guides students to choose. The student thinks through the options and chooses the test that will confirm the diagnosis to feedback to the interactive video. The interactive video pushes the test results and further suggests treatment points and measures. After answering some questions in the interactive video, multiple-choice questions on knowledge related to peptic ulcers are hidden. Students will input the selected answers into the test system set up by the online teaching software (Learning Link), and the teacher can check the students’ work in the background.
(2) Summarizing and reporting stage 1 h: after completing the interactive video, students engage in group discussions to analyze the cases and address any problems that arise. The teacher facilitates the discussion, summarizes the results, and evaluates the overall performance of the group. Teachers guide students through the complexities of the disease and help them extract the main information. The teacher then provides targeted explanations and theoretical foundations to support the students’ analyses. Teachers in this group were validated based on their teaching experience and their proficiency in using the interactive video system. The students in this group worked collaboratively in groups of 8 students each, ensuring active participation and knowledge sharing. A total of 32 students participated in the intervention group, under the guidance of 1 teacher.

#### 2.5.3 Control group

(1) Teaching phase 1 h: classroom teaching method is adopted. The teacher explains the chapter of peptic ulcer in internal medicine based on a similar case before class, focusing on the classification of the disease, laboratory and auxiliary tests, diagnosis, and treatment.
(2) Summarizing and reporting 1 h: similar to the intervention group, students in the control group discuss and report cases and problems in groups. The teacher summarizes the results of the discussion and evaluates the overall performance, providing targeted feedback and emphasizing key aspects of the consultation. The students in this group were also encouraged to collaborate in small groups of 8 students each. A total of 32 students participated in the control group, guided by 1 teacher.

### 2.6 Outcomes

While all outcomes except the Client satisfaction questionnaire-3 (CSQ-3) were measured using a pre-test and post-test., the CSQ-3 was only measured using a post-test. The pre-test was administered before the start of the teaching phase, while the post-test was conducted after the completion of the summary phase.

#### 2.6.1 Primary outcomes

##### 2.6.1.1 Basic knowledge test

The basic knowledge assessment consists of two parts: pre-test and post-test. Each part contains 30 multiple-choice questions, with a maximum score of 30 points. The test content aligns with the standards of the Chinese Medicine Qualification Examination, covering the etiology, staging, clinical symptoms, diagnosis, treatment, and prognosis of colorectal cancer.

Once the test items are completed, they will be reviewed by a multidisciplinary expert team from fields such as preventive medicine, primary healthcare medicine, medical psychology, and gastroenterology. The final selection of pre- and post-test questions will be made by two co-directors to ensure the difficulty level of both sets of tests is consistent, maintaining the integrity and fairness of the assessment.

#### 2.6.2 Secondary outcomes

##### 2.6.2.1 Clinical Thinking Ability Assessment Scale

This study uses the Medical Student Clinical Thinking Ability Assessment Scale developed by Zhong-yan (29). The scale was designed based on a survey of factors influencing clinical thinking ability, and includes three evaluation dimensions: critical thinking ability, with 6 items; systematic thinking ability, with 11 items; and evidence-based thinking ability, with 7 items. The scale consists of 24 items in total to assess the clinical thinking ability of medical students. In Song Junyan’s study, the internal consistency reliability of the scale was 0.909, and the content validity of each item ranged from 0.75 to 1.00. In this study, the Cronbach’s α coefficient of the questionnaire is 0.966.

##### 2.6.2.2 Course Experience Questionnaire-28

This questionnaire was developed by Ramsden et al. (30). It is widely used in Australia for the quality assessment of higher education. Based on previous work, Peng and others translated and revised the Chinese version of the CEQ-28 scale. The scale includes four dimensions: good teaching, reasonable workload, quality of teaching, and learning outcomes. The internal consistency reliability of the questionnaire is 0.928, indicating high stability and reliability (31). In this study, the Cronbach’s α coefficient of the questionnaire is 0.960.

##### 2.6.2.3 Client Satisfaction Questionnaire-3

The CSQ-3 includes three items to evaluate participants’ satisfaction with the decision tree-based interactive video on the diagnosis and treatment of peptic ulcers. The CSQ-3 uses a 4-point Likert scale, with scores ranging from 1 (low satisfaction) to 4 (high satisfaction). Since the study participants differ from those in the original questionnaire, modifications were made to the subject of the questionnaire while other content remained unchanged. In this study, the Cronbach’s α coefficient for this questionnaire is 0.907.

### 2.7 Statistic

The study was statistically analyzed using SPSS Statistics 27.0 software. Continuous variables were expressed as mean ± standard deviation (X ± S) and categorical variables were expressed as numbers and percentages (frequency %). For continuous data, a normality test was first performed. If the groups conformed to normality and the variance between the two groups was equal, the *t*-test was used for between-group comparisons; otherwise, the nonparametric Wilcoxon rank sum test was used. For categorical data, the chi-square test was used for unordered results and the nonparametric Wilcoxon rank sum test was used for ordinal data. All statistical tests were performed using two-tailed tests, and the corresponding *P*-values were reported. Hypothesis testing for the primary outcome indicators used a significance level of α = 0.05. We considered differences to be statistically significant when the *P*-value was less than 0.05.

## 3 Results

### 3.1 Demographic characteristics

A total of 64 students were initially recruited for this study, with 32 students allocated to the intervention group and 32 to the control group. However, two students in the control group did not complete the study, leaving 62 participants for the final analysis. The mean age of the participants was 22.53 years, and the majority were female (62.90%, *n* = 39). Most participants identified as Han ethnicity (90.32%, *n* = 56), and 75.81% (*n* = 47) had a rural household registration. In terms of academic performance, 50% (*n* = 31) of the students ranked between the 31st and 60th percentiles in school performance. The majority (64.52%, *n* = 40) reported a monthly living allowance between 801 and 1,500 RMB, while 40.32% (*n* = 25) had a family monthly income exceeding 5000 RMB. Regarding parental education, most fathers (53.23%, *n* = 33) and mothers (54.84%, *n* = 34) had received secondary education. Additionally, 85.48% (*n* = 53) of the participants reported having a medical background. Baseline demographic characteristics were generally well balanced between the intervention and control groups, except for a significant difference in the gender distribution (Table 1).

**TABLE 1:** Comparison of baseline demographic characteristics of students.

Variables	Total (*n* = 62)	Control group (*n* = 30)	Intervention group (*n* = 32)	*P*
Age, mean ± SD	22.53 ± 0.74	22.50 ± 0.78	22.56 ± 0.72	0.743
Sex, n (%)				0.007
Male	23 (37.10)	6 (20.00)	17 (53.12)	
Female	39 (62.90)	24 (80.00)	15 (46.88)	
Nation, n (%)				1.000
Han	56 (90.32)	27 (90.00)	29 (90.62)	
Minority	6 (9.68)	3 (10.00)	3 (9.38)	
Hukou, n (%)				0.455
Non-agriculture	15 (24.19)	6 (20.00)	9 (28.12)	
Agriculture	47 (75.81)	24 (80.00)	23 (71.88)	
Top percentage of school performance, n (%)				0.762
≤ 30	16 (25.81)	9 (30.00)	7 (21.88)	
31-60	31 (50.00)	14 (46.67)	17 (53.12)	
>60	15 (24.19)	7 (23.33)	8 (25.00)	
Monthly living expenses (CNY), n (%)				1.000
≤ 800	7 (11.29)	3 (10.00)	4 (12.50)	
801-1,500	40 (64.52)	20 (66.67)	20 (62.50)	
1,501-2,000	10 (16.13)	5 (16.67)	5 (15.62)	
2,001-2,500	4 (6.45)	2 (6.67)	2 (6.25)	
≥ 2,501	1 (1.61)	0 (0.00)	1 (3.12)	
Per capita monthly household income (CNY), n (%)				0.305
≤ 3,000	15 (24.19)	5 (16.67)	10 (31.25)	
3,001-5,000	22 (35.48)	13 (43.33)	9 (28.12)	
>5,000	25 (40.32)	12 (40.00)	13 (40.62)	
Father’s education background, n (%)				0.315
Primary education	15 (24.19)	9 (30.00)	6 (18.75)	
Second education	33 (53.23)	13 (43.33)	20 (62.50)	
Higher education	14 (22.58)	8 (26.67)	6 (18.75)	
Mother’s education background, n (%)				0.727
Primary education	16 (25.81)	9 (30.00)	7 (21.88)	
Second education	34 (54.84)	16 (53.33)	18 (56.25)	
Higher education	12 (19.35)	5 (16.67)	7 (21.88)	
Whether the parents or relatives are engaged in clinical medicine related majors, n (%)				0.537
Yes	9 (14.52)	3 (10.00)	6 (18.75)	
No	53 (85.48)	27 (90.00)	26 (81.25)	

### 3.2 Basic knowledge test

**FIGURE 2:**
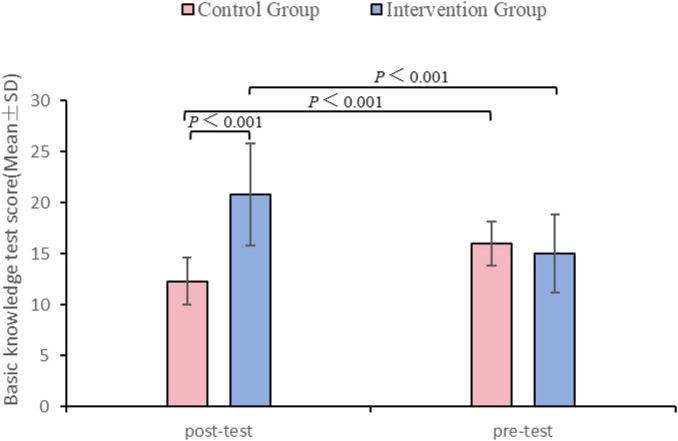
Basic knowledge test scores: pre-test and post-test comparison between control and intervention groups.

There was no significant difference in the basic knowledge test scores between the two groups before teaching (Table 2 and Figure 2). After teaching, the test scores of intervention group showed a statistically significant improvement compared with the control group (*P* < 0.001), and the scores of the intervention group were also significantly higher than the baseline scores (*P* < 0.001) (Tables 2, 3). However, our study unexpectedly found that the test scores of the control group showed a significant decline compared to their baseline scores after the intervention (*P* < 0.001).

**TABLE 2:** Comparison of the basic knowledge test scores in control and intervention groups.

Variable	Control group	Intervention group	*t*	*P*
Pre-test, Mean ± SD	16.00 ± 2.15	15.00 ± 3.78	–1.29	0.203
Post-test, Mean ± SD	12.27 ± 2.30	20.81 ± 4.98	8.76	<0.001

SD, standard deviation; t, *t*-test.

**TABLE 3:** Comparison of the basic knowledge test scores in pre-and post-test.

Variable	Pre-test	Post-test	*t*	*P*
Control group, Mean ± SD	16.00 ± 2.15	12.27 ± 2.30	–6.49	<0.001
Intervention group, Mean ± SD	15.00 ± 3.78	20.81 ± 4.98	5.26	<0.001

SD, standard deviation; t, *t*-test.

### 3.3 Clinical thinking ability

**FIGURE 3:**
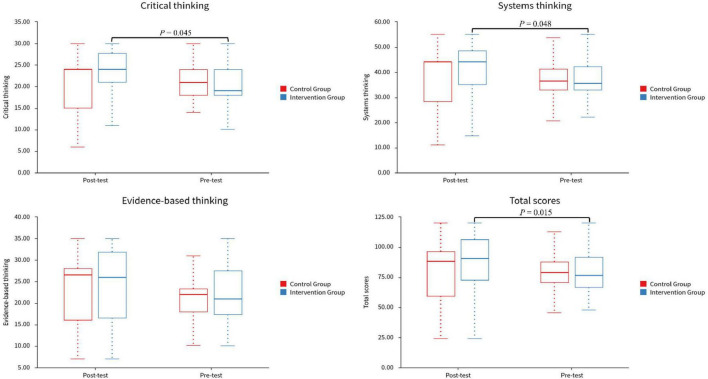
Pre-test and post-test comparison of clinical thinking between control and intervention groups.

The post-test scores of the intervention group in critical thinking (*P* = 0.037) and systematic thinking (*P* = 0.045) were significantly higher than those in the pre-test, while the control group showed no significant changes in these two dimensions (Table 4 and Figure 3). Evidence-based thinking also did not change significantly between the two groups. For the total scores, the post-test of intervention group showed a significant improvement compared to the baseline (*P* = 0.015). In the pre and post-test, the two groups showed no significant differences in each dimension and total scores (Table 5 and Figure 3).

**TABLE 4:** Intragroup comparison of pre-and post-test outcomes for control and intervention groups.

Variable	Control group						Intervention group					
	**Pre-test**		**Post-test**		**Statistic**	***P***	**Pre-test**		**Post-test**		**Statistic**	***P***
	**M (Q*1*, Q*3*)**	**Mean**	**M (Q*1*, Q*3*)**	**Mean**			**M (Q*1*, Q*3*)**	**Mean**	**M (Q*1*, Q*3*)**	**Mean**		
Critical thinking ability	21.00 (18.25, 23.75)	21.10	24.00 (15.00, 24.00)	20.30	Z = 0.491	0.624	19.00 (18.00, 24.00)	21.03	24.00 (21.00, 27.25)	23.00	Z = 2.008	0.045
Systems thinking ability	36.50 (33.00, 41.00)	37.10	44.00 (29.75, 44.00)	38.10	Z = 0.278	0.781	35.50 (33.00, 40.75)	36.97	44.00 (35.00, 47.50)	39.91	Z = 1.979	0.048
Evidence-based thinking ability	22.00 (18.75, 23.00)	21.27	26.50 (16.75, 28.00)	23.03	Z = 0.992	0.321	21.00 (17.75, 26.50)	22.50	26.00 (17.50, 31.25)	23.72	Z = 1.157	0.247
Total scores	79.00 (71.00, 86.50)	79.47	88.00 (61.25, 96.00)	81.43	t = 0.439	0.664	76.50 (69.50, 87.25)	80.50	90.50 (74.25, 106.00)	86.62	Z = 2.439	0.015

M, Median; Q*1*, 1st Quartile; Q*3*, 3st Quartile; Z, Wilcoxon Signed-Rank Test; t, *t*-test.

**TABLE 5:** Comparison of pre-test and post-test of clinical thinking ability in control and intervention groups.

Variable	Pre-test						Post-test					
	**Control Group**		**Intervention Group**		**Statistic**	***P***	**Control Group**		**Intervention Group**		**Statistic**	***P***
	**M (Q*1*, Q*3*)**	**Mean**	**M (Q*1*, Q*3*)**	**Mean**			**M (Q*1*, Q*3*)**	**Mean**	**M (Q*1*, Q*3*)**	**Mean**		
Critical thinking ability	21.00 (18.25, 23.75)	21.10	19.00 (18.00, 24.00)	21.03	Z = –0.37	0.712	24.00 (15.00, 24.00)	20.30	24.00 (21.00, 27.25)	23.00	Z = –1.88	0.060
Systematic thinking ability	36.50 (33.00, 41.00)	37.10	35.50 (33.00, 40.75)	36.97	Z = –0.35	0.724	44.00 (29.75, 44.00)	38.10	44.00 (35.00, 47.50)	39.91	Z = –0.83	0.405
Evidence-based thinking ability	22.00 (18.75, 23.00)	21.27	21.00 (17.75, 26.50)	22.50	Z = –0.16	0.876	26.50 (16.75, 28.00)	23.03	26.00 (17.50, 31.25)	23.72	Z = –0.50	0.618
Total scores	79.00 (71.00, 86.50)	79.47	76.50 (69.50, 87.25)	80.50	Z = –0.27	0.783	88.00 (61.25, 96.00)	81.43	90.50 (74.25, 106.00)	86.62	Z = –1.05	0.296

M, Median; Q*1*, 1st Quartile; Q*3*, 3st Quartile; Z, Mann-Whitney test.

### 3.4 Course experience

**FIGURE 4:**
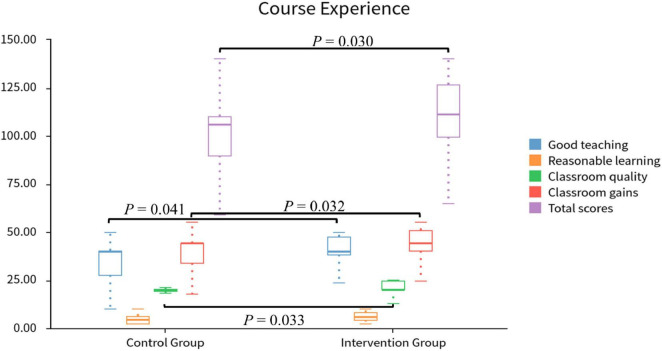
Comparison of course experience between control and intervention groups.

The results showed that the intervention group was significantly higher than the control group in three aspects (Table 6 and Figure 4): good teaching (*P* = 0.041), classroom quality (*P* = 0.033), and classroom gains (*P* = 0.032). However, in terms of reasonable learning load, there was no difference between the two groups (*P* = 0.130). The total scores also showed significant improvement in the intervention group (*P* = 0.030).

**TABLE 6:** Comparison of course experience in control and intervention groups.

Variable	Control group		Intervention group		Statistic	*P*
	**M (Q*1*, Q*3*)**	**Mean**	**M (Q*1*, Q*3*)**	**Mean**		
Good teaching	40.00 (28.75, 40.00)	35.17	40.00 (38.00, 46.50)	40.25	Z = –2.04	0.041
Reasonable learning load	4.50 (2.00, 6.00)	4.67	6.00 (4.00, 8.00)	5.72	Z = –1.51	0.130
Classroom quality	20.00 (20.00, 20.00)	18.67	20.00 (20.00, 24.25)	20.84	Z = –2.13	0.033
Classroom gains	44.00 (36.50, 44.00)	39.37	44.00 (42.75, 50.25)	44.16	Z = –2.14	0.032
Total scores	106.00 (93.00, 110.00)	97.87	111.00 (99.75, 122.75)	110.97	Z = –2.17	0.030

M, Median; Q*1*, 1st Quartile; Q*3*, 3st Quartile; Z, Mann-Whitney test.

### 3.5 Clients’ satisfaction

The results showed that the intervention group was significantly higher than the control group in meeting learning needs (*P* = 0.026), reusability (*P* = 0.004) and total score (*P* = 0.011) (Table 7). In terms of satisfaction, the intervention group’s score also improved to a certain extent. However, statistical significance was not reached (*P* = 0.051).

**TABLE 7:** Comparison of the satisfaction scores in control and intervention groups.

Variable	Control group		Intervention group		Statistic	*P*
	**M (Q*1*, Q*3*)**	**Mean**	**M (Q*1*, Q*3*)**	**Mean**		
Learning needs	2.00 (2.00, 2.00)	2.10	2.00 (2.00, 3.00)	2.50	Z = –2.23	0.026
Satisfaction	2.00 (2.00, 2.00)	2.13	2.00 (2.00, 3.00)	2.41	Z = –1.95	0.051
Reusability	2.00 (2.00, 2.00)	2.10	3.00 (2.00, 3.00)	2.50	Z = –2.86	0.004
Total scores	6.00 (6.00, 6.00)	6.30	7.50 (6.00, 9.00)	7.28	Z = –2.53	0.011

M, Median; Q*1*, 1st Quartile; Q*3*, 3st Quartile; Z, Mann-Whitney test.

## 4 Discussion

The study results revealed that interactive video-based CBL presented distinct advantages over traditional CBL in the context of clinical medical education. The intervention group exhibited a statistically significant improvement in basic knowledge test scores following the teaching intervention, both in comparison to their baseline scores and to the control group. This finding indicates that the interactive video format is highly effective in enhancing knowledge acquisition and retention (32, 33). Conversely, the control group experienced a significant decline in test scores post-intervention, an unexpected outcome that suggests potential challenges in sustaining learning outcomes with traditional CBL methods. This decline may be attributed to factors such as reduced engagement, limited interaction, or insufficient reinforcement of key concepts during traditional teaching sessions.

The theoretical basis for the success of the interactive video-based CBL method can be supported by Cognitive Load Theory, which emphasizes the importance of managing cognitive load in learning environments (34). Interactive videos allow learners to control the pace of their learning, providing an environment where they can process information actively through engagement with the content, which in turn reduces extraneous cognitive load and facilitates deeper processing. This aligns with Multimedia Learning Theory, which suggests that learners are more likely to retain information when it is presented in both visual and auditory formats, especially when they can interact with the content (35). The integration of decision trees and scenario-based questions in the interactive video further supports this theory by prompting learners to actively engage with the content, thereby enhancing their understanding and retention.

The intervention group exhibited significant improvements in both critical thinking and systematic thinking abilities, underscoring the effectiveness of the interactive video format in enhancing clinical reasoning skills. This finding is consistent with the study by Lim and Veasuvalingam (32), which highlighted the benefits of online case-based learning (CBL) in supporting the development of clinical reasoning skills among medical students (32). Their study found that online CBL facilitated improved question exploration, immediate feedback, and the opportunity for in-depth discussions during virtual consultations, all of which contributed to enhanced clinical reasoning. However, the present study goes a step further by integrating interactive video into the online CBL format, which appears to have a more pronounced effect on learning outcomes. This aligns with Zhang et al. (33), who demonstrated that students in e-learning environments with interactive video achieved significantly better performance and greater satisfaction compared to those in non-interactive video or traditional classroom settings. The inclusion of interactivity in instructional video enhances learning effectiveness and learner engagement, reinforcing the idea that interactive video can be a powerful tool in modern educational environments (36).

However, no notable changes were observed in evidence-based thinking within either group. This suggests an opportunity for further optimization of the interactive video-based case-based learning (CBL) method, potentially through the incorporation of modules specifically designed to bolster evidence-based decision-making processes. The findings regarding clinical thinking abilities are consistent with prior research that emphasizes the importance of active learning and scenario-based teaching in fostering deeper cognitive engagement and analytical skills. For example, Huang et al. (37) evaluated the effectiveness of team-, case-, lecture-, and evidence-based learning (TCLEBL) in medical postgraduate training and found that the TCLEBL approach led to significantly better outcomes compared to traditional lecture-based learning (LBL). Students in the TCLEBL group demonstrated superior performance in theoretical tests and literature reviews, particularly in terms of scientific rigor, argumentation, and the incorporation of evidence-based practices. These findings align with the suggestion that integrating evidence-based learning components into the interactive video-based CBL method could enhance the development of evidence-based thinking alongside clinical reasoning. The study by Huang et al. (37) further highlights the importance of actively engaging students in evidence-based decision-making, which could be achieved through targeted curriculum optimization in future iterations of the interactive video CBL approach.

In addition to learning load, the evaluation of the course experience indicated that the intervention group rated their learning experience significantly higher regarding teaching quality, classroom environment, and perceived learning gains. Meanwhile, the intervention group showed significantly higher course satisfaction compared to the control group. These findings highlight the potential of interactive video-based teaching to enhance student satisfaction and engagement, especially in online or hybrid learning environments where traditional methods may be less effective. A study by Natarajan et al. (38) comparing an interactive educational video-based strategy with traditional demonstration-based teaching in nursing found that, while both approaches showed similar knowledge and skill competency scores, the interactive video approach resulted in higher satisfaction levels among students, with 92% of students expressing satisfaction with the video learning method (38). This aligns with the positive impact of interactive video on student engagement and learning satisfaction in the context of nursing education, reinforcing the value of this method in enhancing the learning experience. Additionally, a study by Dong (19) analyzing student feedback on interactive video-based teaching also emphasized the critical role of interactivity, content quality, and technical support in improving student satisfaction (19). Furthermore, a study by Schaffner and Vogt (39) exploring the use of interactive technology in advanced pharmacology courses revealed positive outcomes in both knowledge acquisition and student satisfaction (39). The use of evolving case studies coupled with interactive technology, such as blogs and wikis, was shown to foster critical thinking, clinical reasoning, and increased interaction among students, leading to higher satisfaction levels. These findings further support the notion that interactive video-based teaching is an effective method for enhancing student engagement, satisfaction, and learning outcomes.

The significant decline in performance observed in the control group is an important finding that warrants further investigation. Traditional CBL methods, while effective in small-scale or face-to-face settings, may struggle to maintain student motivation and engagement in larger or online formats. This is consistent with the work of Telner et al. (40), who pointed out that traditional CBL often lacks the interactive and engaging elements that are necessary to keep students motivated, particularly in remote or hybrid learning environments (40). The results of the study suggest that traditional CBL needs to be adapted to better suit the needs of modern learners who are accustomed to interactive and dynamic digital environments (38, 41).

## 5 Limitation

Despite the promising results, this study has several limitations. The sample size was relatively small and drawn from a single institution, which limits the generalizability of the findings. Furthermore, the study concentrated on short-term outcomes, leaving the long-term effects of interactive video-based CBL on knowledge retention and clinical thinking unassessed. The significant decline in the scores of the control group also raises questions about potential confounding factors, such as external influences or variations in student motivation, which were not controlled for in this study.

## 6 Future

Future research should aim to address these limitations by expanding the sample size, incorporating multiple institutions, and examining the long-term impacts of interactive video-based case-based learning (CBL). Additionally, refining the design of interactive videos to include more evidence-based content and adaptive learning pathways may further enhance their effectiveness. Understanding the underlying factors contributing to the decline in the performance of the control group, such as student motivation, external influences, and variations in engagement, will also be critical for improving traditional online CBL methods and ensuring equitable learning outcomes across various teaching modalities. Furthermore, future studies could compare the efficacy of interactive video-based CBL teaching without teacher/facilitator support to traditional CBL methods, as this would provide deeper insights into the impact of teacher involvement on learning outcomes. These efforts will contribute to establishing interactive video-based CBL as a transformative approach in clinical medical education.

## 7 Conclusion

This study evaluated the effectiveness of an interactive video-based decision tree teaching method in enhancing clinical knowledge, critical thinking, and systematic thinking skills among medical students. The results showed that the interactive video-based teaching significantly improved students’ foundational knowledge and cognitive skills, particularly in critical and systematic thinking. Additionally, the intervention group reported significantly higher scores in course experience and overall satisfaction, suggesting that the interactive video teaching method not only improved academic outcomes but also increased student engagement and satisfaction with the course. Overall, this study demonstrates that the interactive video-based decision tree teaching method is an effective tool for improving clinical education, particularly in enhancing student participation, learning outcomes, and cognitive skills. Given its potential for large-scale and online education settings, it could serve as an innovative teaching approach to complement traditional methods in medical education. Future research should further explore its applicability to other disciplines and its long-term effects.

## Data Availability

The raw data supporting the conclusions of this article will be made available by the authors, without undue reservation.

## Footnotes

1
https://www.bilibili.com
